# Compound events and associated impacts in China

**DOI:** 10.1016/j.isci.2022.104689

**Published:** 2022-06-30

**Authors:** Zengchao Hao

**Affiliations:** 1 College of Water Sciences, Beijing Normal University, Beijing, 100875, China

**Keywords:** Earth sciences, Integrated geography, Climatology, Hydrology

## Abstract

Owing to amplified impacts on human society and ecosystems, compound events (or extremes) have attracted ample attention in recent decades. China is particularly vulnerable to compound events due to the fast warming rate, dense populations, and fragile ecological environment. Recent studies have demonstrated tangible effects of climate change on compound events with mounting impacts on the economy, agriculture, public health, and infrastructure in China, posing unprecedented threats that are increasingly difficult to manage. Here, I synthesize recent progress in studies of compound events and associated impacts in China. Several lines of evidence indicate an increase in the frequency and intensity of multiple types of compound events across China. Future directions in studying compound events in China are suggested, including investigating extremes from a compound perspective, modeling compound events in the Anthropocene, quantitative evaluations of risks, and holistic adaptation measures of compound events.

## Graphical abstract

**Figure:**
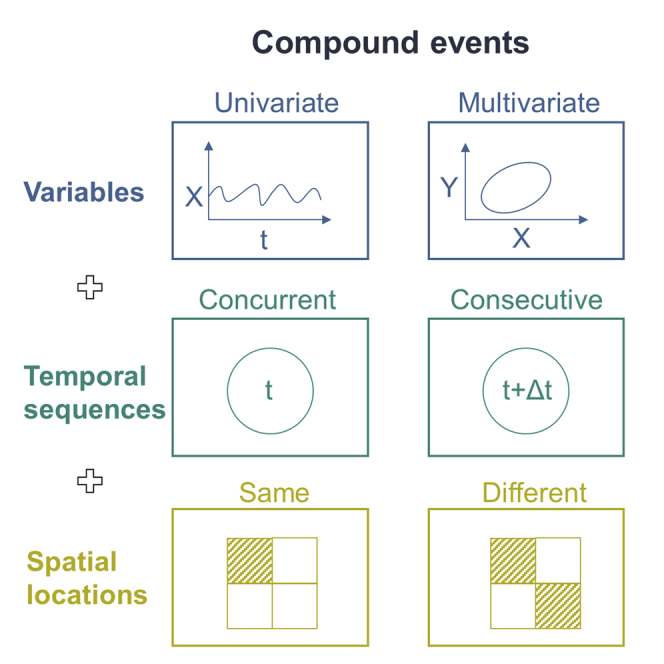

## Introduction

China has been gripped not only by individual weather and climate extremes (e.g., droughts, heatwaves, and flooding) (Zhao, 2020) but also by concurrences of these extremes in recent decades. The combination of multiple events leading to high social or environmental impacts is often termed compound events or extremes (with multiple types such as preconditioned, multivariate, temporally compounding, and spatially compounding) (Zscheischler et al., 2020), which may induce larger negative impacts on different systems or sectors than their univariate counterparts (Bevacqua et al., 2021; Hao et al., 2013; Raymond et al., 2020; Zscheischler et al., 2018). The past decade has witnessed the burgeoning development in studying compound events in China. Under global warming, increased occurrences of compound events pose serious threats to the economy, human health, and ecosystems across the country, due to complicated climate types (e.g., giving rise to multiple natural disasters) and fast warming rate (e.g., triggering more frequent weather and climate extremes). Moreover, a wide range of changes in human systems (e.g., fast urbanization and increasing populations) and ecosystems (e.g., afforestation and reforestation, changed farming practices) also complicate the impacts of compound events on different regions, sectors, and systems of China (Chen et al., 2019a; Li et al., 2021a). The bulk of studies have projected an increase in many types of compound events in China in the future. There is thus an urgent need to understand compound events and their impacts across the nation under a changing climate.

## Progress of compound event studies

**Figure 1:**
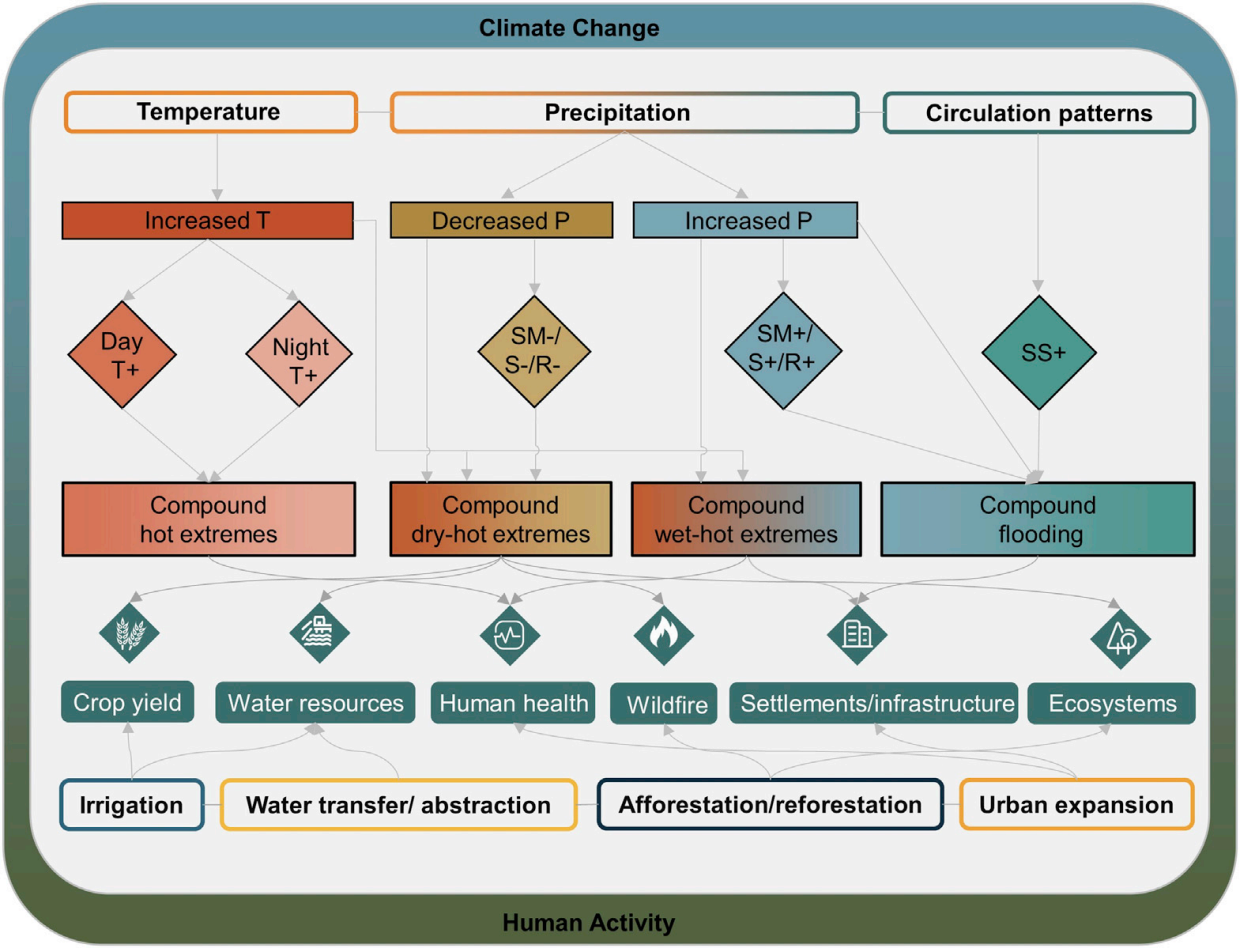
Schematic of several compound events and their potential impacts Temperature, precipitation, soil moisture, streamflow, runoff, and storm surge are abbreviated as T, P, SM, S, R, and SS, respectively. + and - indicate positive and negative anomalies of contributing variables, respectively.

A variety of compound events have been evaluated in China, including compound hot extremes, compound dry-hot extremes, compound wet-hot extremes, and compound flooding, as shown in Figure 1. These events are defined based on the combination of contributing variables of different time scales, which can be investigated by statistical approaches (e.g., empirical counting, indicator approach, and multivariate distribution), dynamical modeling approaches (e.g., climate model ensemble and hydrological model), and socio-physical approaches (e.g., event-based storylines) (Bevacqua et al., 2021; Hao and Singh, 2020; Raymond et al., 2020; Zhang et al., 2021). Their impacts span multiple sectors, including ecosystems, water resources, agricultural production, human health, and infrastructure, which have been documented around the world. In the following, discussions are confined to the main progress in compound event studies in China.

### Compound hot extremes

Compound hot extremes are commonly defined as combined daytime-nighttime hot extremes (Chen and Zhai, 2017; Chen et al., 2019d). The daytime temperature extremes may cause human thermal discomfort or health risks (e.g., causing morbidity and mortality), which, if combined with extreme hot nights, can impair the thermoregulation of human bodies and induce amplified impacts on public health (i.e., a double-strike of hot days and hot nights leading to higher mortality risk) (Chen and Zhai, 2017; He et al., 2021). A recent analysis of major urban agglomerations of China provides evidence that the relative risk of compound hot extremes is higher than that of normal days (daytime or nighttime hot extremes) with females and older groups of city dwellers being more vulnerable (Wang et al., 2021). These compound hot extremes are generally associated with anomalous anticyclones conditions (i.e., resulting in descending motion associated with strong adiabatic heating and more solar radiation) and moisture transport (Chen and Li, 2017; Li et al., 2021c; Ma et al., 2022).

Compound daytime-nighttime hot extremes have become more frequent and longer in China. Owing to the urban heat island (UHI) effect associated with increased urbanization (Shi et al., 2021; Sun et al., 2019), a larger increase in the frequency and duration of compound hot extremes has been shown in urban areas in China (An and Zuo, 2021; Liao et al., 2021a; Wang et al., 2020a). Urbanization was found to contribute to around 40% of the increased frequency and fraction of compound hot extremes in major urban agglomerations of China during 1961–2017 (Wu et al., 2021b). The anthropogenic influence was shown to contribute remarkably to the increase of compound hot extremes across the country. For the increase in urban compound hot extremes from 1961 to 2014 in China (around 1.76 days per decade), the attributable fractions were estimated to be 0.51 and 1.63 days per decade from urbanization and greenhouse gas emissions, respectively (Wang et al., 2021).

Projections of compound hot extremes reveal an increase in the future, with a higher risk in urban areas (Liao et al., 2021a; Su and Dong, 2019; Wang et al., 2020b; Xie et al., 2022). Under the representative concentration pathway (RCP) 8.5 (a high greenhouse gas emissions pathway), almost 50% of land areas in three urban agglomerations of China, including the Beijing-Tianjin-Hebei region (midlatitudes), the Yangtze River Delta (subtropical regions), and the Pearl River Delta (tropical regions), will experience historically unprecedented compound hot extremes regularly by 2050, 2050, and 2030, respectively, with a faster growth rate of the magnitude in Pearl River Delta (Wang et al., 2020b). The high population density in urban areas in China and the continuous increase in the population can further amplify the risk of compound hot extremes. Consequently, the projected increase in the hazard of compound hot extremes will add to the population exposure or risk, especially for females and elderly people (He et al., 2021). These results call for improved understanding and urgent adaptation efforts of compound hot extremes for the rapidly urbanized and highly populated areas, especially eastern China.

### Compound dry-hot extremes

The concurrent or consecutive occurrences of droughts (e.g., due to deficit in precipitation, soil moisture, runoff, streamflow, or groundwater over an extended period) and hot extremes are often termed compound dry-hot extremes (or hot droughts, warm droughts). They may induce amplified impacts on agriculture, ecosystems, and water resources, which can be higher than that from individual droughts or hot extremes. Stationary anticyclones (e.g., blocking, associated with enhanced adiabatic heating through increased subsidence), land-atmosphere feedbacks (associated with soil moisture impacts on surface temperature), and large-scale modes of variability (e.g., El Niño-Southern Oscillation, or ENSO for short) can induce the negative dependence between droughts and hot extremes, resulting in the concurrence of the two extremes of different time scales (Hao and Singh, 2020; Mukherjee et al., 2020; Zscheischler et al., 2020). These driving factors have been shown to contribute to the occurrence of compound dry-hot extremes in China (Li et al., 2020b; Wu et al., 2021c).

In the past 60 years, an increase in the frequency, duration, and spatial extent of compound dry-hot extremes is observed in China, especially after the 1990s (Chen et al., 2019b; Yu and Zhai, 2020b; Zhang et al., 2022), which is accompanied by a remarkable increase in the population exposure (Feng et al., 2021; Wu et al., 2021d). For example, the frequency of compound dry-hot extremes during summer in the densely populated areas of eastern China increases with a significant trend of 0.4 days per decade for the period from 1961 to 2018, and the population exposure to compound dry-hot extremes has doubled since the late 1990s (Yu and Zhai, 2020a). These increases are commonly associated with changed circulation patterns (e.g., increased geopotential height) or enhanced land-atmosphere interactions, as well as anthropogenic influences (Li et al., 2020a, 2021b; Zhang et al., 2020). A consistent increase of compound dry-hot extremes across China is projected in the future (Sun et al., 2017; Zhou and Liu, 2018), and holding global warming at 1.5°C instead of 2°C can reduce around 25% of the spatial extent of compound dry-hot extremes in China (Wu et al., 2021d). Substantial progress has been made in the change detection, attribution, and projections of compound dry-hot extremes in China while quantitative assessments of the impacts and risks on multiple sectors or systems are rather limited.

### Compound wet-hot extremes

Compound wet-hot extremes are usually defined as the consecutive or sequential occurrences of wet conditions (e.g., humidity, high precipitation, and flood) and hot extremes over a specified period. The combined impacts of high temperature and humidity on human discomfort or health have been well recognized (Fischer and Knutti, 2013; Zhang et al., 2021; Zscheischler et al., 2020). Owing to space limitations, we focus on the wet conditions based on precipitation or flood in defining compound wet-hot extremes in the following sections.

The occurrence of hot extremes followed by heavy rainfall with some lags (e.g., 1, 3, and 7 days) is one type of compound events (also referred to as compound heat-precipitation events). The weather or climate conditions of heatwaves can favor the occurrence of heavy precipitation. Specifically, high surface temperature and sensible heat flux associated with heatwaves can enhance the convective available potential energy (CAPE) and provides favorable conditions for the development and maintenance of heavy precipitation (e.g., enhanced atmospheric instability and moisture sources), which, combined with alternatively anomalous anticyclones and cyclones (e.g., in southern China), can produce the consecutive occurrences of heat and extreme precipitation (Ning et al., 2022; Wu et al., 2021a; You and Wang, 2021). Since the 1960s, the fraction of summer extreme precipitation events preceded by heat events within 3 days shows a significant increase of 2.51% per decade (national mean) in most areas of China (Ning et al., 2022).

The sequential occurrences of flood events followed by heatwaves within a prescribed temporal interval (e.g., 7 days) are a newly developed compound wet-hot event recently. Their underlying negative impacts result from the fact that after the blackout caused by flooding, a higher vulnerability of human health to following hot extremes can be induced, as witnessed during the sequential flood-heatwaves in July 2018 in Japan (Chen et al., 2021; Wang et al., 2019). In southern China, the recurrent arrivals of tropical intraseasonal oscillations and subtropical jets can result in the sequential occurrence of floods and heatwaves and the atmospheric memory of passages of tropical cyclones can also induce heatwaves after a flood (Chen et al., 2021). The occurrence of sequential flood-heatwave events has increased remarkably in recent decades due to significantly increased heatwaves. Specifically, for the period from 1961 to 2018, the probability of compound flood-heatwave events after the 2000s is 5–10 times the level in the 1960s in southern, northwestern, and northeastern China (Chen et al., 2021).

These two types of compound wet-hot extremes are shown to increase in future periods. Projections from climate models indicate a significantly increasing trend in the frequency of compound heat-precipitation events by the end of the 21st century (increase by 1- to 5-times) over most regions of China (You and Wang, 2021). In addition, the frequency and spatial extent of sequential flood-hot extremes are projected to increase by 2100 (e.g., more than 90% of the country is exposed to this event), with earlier emergence in southwest and southeast China (Liao et al., 2021b). These results imply high risks of compound wet-hot extremes to human health and infrastructure in the future.

### Compound flooding

Floods in coastal regions are related to the extreme sea level (including storm surges, astronomical tides, and waves), river discharge, and surface rainfall-runoff (i.e., coastal, fluvial, and pluvial floods) (Fang et al., 2021; Hendry et al., 2019). Compound flooding in coastal areas is commonly defined as the concurrences of anomalies of hydrometeorological variables (e.g., precipitation, surface runoff, river discharge, or streamflow) and oceanic variables (e.g., storm surge, tide, wave, and sea level) (Bevacqua et al., 2019; Hao and Singh, 2020; Wahl et al., 2015) leading to flooding events. Coastal flooding risks may be underestimated if only the individual flood is considered (Liu et al., 2022; Wahl et al., 2015). Early studies have evaluated the joint impact of rainfall and tidal level based on the copula model in several coastal cities of China (Lu et al., 2022), such as Fuzhou (Lian et al., 2013) and Haikou (Liu et al., 2022; Xu et al., 2019). Recently, a systematic analysis of the driving factors and impacts of compound flooding along the coast of China was assessed based on long records of precipitation and storm surge from 11 tide gauges (with overall positive dependence between the two contributing variables), in which a higher likelihood of compound flood events was found during the tropical cyclone seasons (Fang et al., 2021). This study also found the important role played by the sea-level rise on a high frequency of compound flood events. In light of continuing/accelerating sea-level rise (Feng et al., 2019a) and the dense populations near coastal areas of China, understanding changes and potential impacts of compound flooding in coastal areas is critical for designing coastal infrastructure under global warming.

## Future directions

### A compound perspective on extremes

The recent focus on compound weather and climate extremes has highlighted the need of viewing extremes through the lens of a compound perspective. A large number of compound events studies in China focus on temperature-related extremes (e.g., increases in hot extreme-dominated compound events in Section 2) at the same location, while compound hydrologic or hydroclimatic extremes of different temporal sequences (e.g., temporally compounding extremes) or spatial locations (e.g., spatially compounding extremes) are rather limited. For example, the precipitation whiplash events (Chen, 2020; Swain et al., 2018) or a rapid transition between floods and droughts (He and Sheffield, 2020), which reflects a volatile hydrological cycle, would challenge water management. Albeit several studies on the transition between floods and droughts in China (Chen et al., 2020), their physical mechanisms and future changes at different time scales are still limited. Other types of compound events, such as concurrent droughts at multiple breadbaskets, have been investigated (e.g., detection, attribution, and projection) in different regions across the globe (Zscheischler and Lehner, 2022; Zscheischler et al., 2020), but are under-assessed in China (Ye and Qian, 2021). Studies on different types of compound events across China are needed for an improved understanding of their characteristics, processes, and risks.

Some hydroclimatic events with large impacts during recent decades in China can also be viewed as compound events, such as sand storms and saltwater intrusions. The severe sand storm in spring 2021 sweeping across northern China (Gui et al., 2021) is a case in point. Sand storms are affected by both surface conditions (e.g., vegetation coverage and soil moisture) and meteorological factors (e.g., wind field and large-scale pressure fields) (Xu et al., 2020). In certain regions, the high temperature and low precipitation are the main reason for sand or dust storms (Dar et al., 2022), which indicates the role played by initial conditions of compound dry-hot extremes. In addition, the combination of extremely low discharges of the Yangtze River and saltwater intrusions during the summer of 2006 in the Yangtze Estuary (Chen et al., 2019c) is a telling example of compound events in China. The concurrences of droughts (climate-induced or human-induced) and salt tides can cause water shortages in cities . Its impacts may be amplified with sea-level rise in the future, heightening a potential risk of coastal regions in China under global warming. Owing to the potentially amplified impacts, diagnosing extremes or risks with multiple contributing variables from a compound event perspective in the coupled meteorological, hydrological, and oceanographic system can aid the understanding of their driving factors and associated impacts.

### Modeling compound events in the Anthropocene

Multiple hydroclimatic factors (e.g., circulation patterns and propagation of hydrological anomalies) and human-related factors (e.g., irrigation and land-use changes) influence occurrences or impacts of compound events (Hao and Singh, 2020; Raymond et al., 2020). The direct human interventions (e.g., water abstractions, reservoirs, and inter-basin water-transfers) and changes in land use/land cover (such as changed farming practices, reforestation, and urbanization) in the human-influenced era can affect water cycles (e.g., droughts) (Van Loon et al., 2016) and regional land hydrology and climate (IPCC, 2022), complicating the understanding of compound extremes in China. For example, owing to the large conversion of rain-fed cropland to irrigated cropland in China (mainly in North China Plain and northeastern China), the water consumption for irrigation in China has increased by 17% for the period from 1987 to 2010 (Zuo et al., 2018). In addition, due to the expansion of natural forests and afforestation, a 19% increase in forest areas during 1999–2013 is found in China based on forest inventories (Chen et al., 2019a). These changes would inevitably affect assessments of compound events and associated impacts on crop yields and ecosystem services. In addition, the urbanization level in China has increased from 35% in 1999 to 56% in 2015 (http://www.stats.gov.cn/). The important role of urbanization in affecting regional weather/climate extremes (Qian et al., 2022) and land hydrology (e.g., an increase of land surface permeability) implies that incorporating urbanization changes is needed in modeling certain compound events (e. g., compound hot extremes and compound flooding) and their impacts. Owing to intense human activities interacting with natural environments in China, modeling the dynamics of key human interventions or land-use practices is paramount for understanding compound event occurrences (or changes) and developing mitigation strategies.

### Quantitative risk assessments of compound events

The collation and synthesis in Section 2 indicate that the impacts of compound events on natural and human systems in China are seldomly quantified. The ultimate impacts of compound events are also related to the exposure and vulnerability of human and natural systems to hydroclimatic hazards. The complex risk (or impact) of compound events can also be high if the exposure or vulnerability is high, though the event may not be extreme. Recent progress of compound events in China indicates that most current studies focus on the hazard aspect (e.g., changes in frequency or intensity). A few studies take a step further in assessing changes in the exposure of croplands or populations of compound events in China (Feng et al., 2021; Wu et al., 2021d). However, the risk assessment (i.e., encompassing hazard, exposure, and vulnerability) of these compound events in different sectors or systems (e.g., agriculture, water resources, and public health) is still limited. For example, concurrent droughts and heatwaves can cripple crop production (Feng et al., 2019b; Li et al., 2022); however, the vulnerability of different crops to this compound extreme is largely under-assessed. Future studies should incorporate hazard, exposure, and vulnerability (or both physical drivers and societal factors) in risk assessments of compound events on human systems (e.g., crop yield, water resources, public health, and infrastructure) and ecosystems (e.g., structure and phenology) in China.

The lack of collaboration between disciplines and sectors (e.g., data sharing and knowledge exchange) also hinders the risk assessments of compound events with influences on multiple sectors that are possibly interconnected. For example, it is challenging for regional climate, hydrology, agriculture, and socioeconomics systems to cover the entire chain of risk analysis (e.g., impacts of compound dry-hot extremes on water or food security), including hazards, exposure, vulnerability, impacts, or risks. Encouragingly, there are interdisciplinary research efforts that employ compound climatic hazards as drivers to perform quantitative risk analysis in other disciplines or communities. For example, the epidemiology community has used compound hot extremes as drivers to quantify mortality risk in China by incorporating the vulnerability of people of different age and gender groups (He et al., 2021; Li et al., 2021d). This calls for enhanced cooperation between disciplines and sectors and integrated actions among stakeholders of different sectors to understand the impacts and risks of compound events.

From the methodological perspective, certain new developments of model simulations and evaluations of compound events have not been widely employed for risk estimation of compound events in China. First, some compound events have an extremely low probability, which makes them difficult to sample and model based on observational records or historical simulations. In addition, compound events are subject to modulation of unforced internal variability, the realizations of which are under-sampled in the Coupled Model Intercomparison Project (CMIP) simulations (Liao et al., 2021b). The single model initial-condition large ensemble (SMILE) (Deser et al., 2020) provides a unique opportunity to sample the low-probability, high-impact compound events and quantify the internal variability in the climate system. Though these large ensemble simulations have been utilized in analyzing compound events in different regions (Bevacqua et al., 2022; Raymond et al., 2022), their usage in compound events analysis in China has been limited (Liao et al., 2021b). Second, the evaluation of model simulations is a key step in risk assessments of compound events based on climate model simulations or projections (Zscheischler and Lehner, 2022). However, assessments of model performances in simulating different types of compound events (and bias correction) with multivariate-based approaches and metrics in China are still limited. In this case, a deeper insight into the underlying dynamic and thermodynamic processes of the occurrences of different compound events is required, which not only helps identify and predict compound events in China but also benefits model evaluation through selecting suitable models and statistics. Overall, more efforts are needed in sampling compound events with large ensemble simulations and evaluating model performances with novel approaches and metrics to aid risk assessments of compound events.

### Holistic adaptation measures of compound events

**Figure 2:**
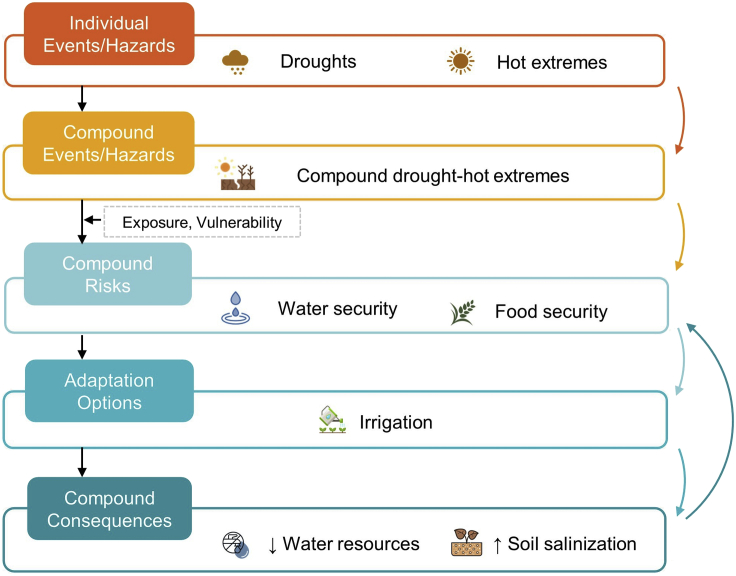
Potentially compound consequences of adaptation measures (i.e., irrigation) for compound risks of water security and food security from compound drought-hot extremes

Whether extremes lead to disasters will also depend on adaptation measures. Climate change and associated extremes have affected different aspects of the society in China (Ding et al., 2021) and multiple strategies of adaptation to individual hazards, such as droughts and hot extremes, have been evaluated in China. However, assessments of hard and soft adaptation options of compound events in China under different global warming levels are still lacking. For example, adaptation options for water security and food security (e.g., irrigation) have been developed for individual extremes (e.g., droughts or heatwaves). However, during compound droughts and heatwaves, the risk of water security and food security may be combined (as shown in Figure 2) (IPCC, 2022). Building on the impact and risk assessment of compound events, evaluating adaptation strategies or options to the impacts on different sectors or systems are needed to cope with the complex risk of compound events.

Meanwhile, there may be compounding consequences of adaptation options to compound risks. In other words, the human adaptation options or response of one risk may increase or decrease the risk of other extremes (Field et al., 2012; Simpson et al., 2021). For example, irrigation (e.g., from groundwater abstraction or reservoir operation) is a useful adaptation measure for addressing the risk of droughts and associated food insecurity (possibly resulting from combined impacts of heatwaves). However, it may lead to reduced groundwater levels and cause reduced water resources (or droughts in other forms or regions, such as downstream), and even soil salinization (due to excess leakage or increased groundwater recharge in irrigated areas) (IPCC, 2022), manifesting a compounding or cascading consequences of an adaptation response, as shown in Figure 2. This can be particularly an issue in northern and northwestern China with limited water resources and existing issues of soil salinization. The need to build resilience to compound extremes warrants holistic adaptation measures of compounding risks for multiple sectors or systems with a transdisciplinary approach, which can aid the adaptation benefits and costs analysis of compound events with the engagement of multi-stakeholders.

### Conclusions

Compound events are related to the occurrences of multiple events (either the same or different variables) at certain temporal sequences (either concurrent or sequential) and/or spatial locations (either single or multiple) leading to impacts on different sectors of natural and human systems, where the compounding effects can be associated with the hazard, exposure, or vulnerability. These events have struck China with disastrous impacts on agriculture, water resources, human health, infrastructure, and ecosystems in recent decades. The past decade has witnessed increasing efforts in investigating diverse types of compound events, including compound hot extremes, compound dry-hot extremes, compound wet-hot extremes, and compound flooding, which covers a wide range of research topics, such as change detection and attribution of observed changes and projection of future changes. However, for different types of compound events in China, diagnosing physical processes of event occurrences, attributing their changes, quantifying risks, and developing adaptation measures are overall under-explored.

Besides improvements in datasets and models for investigating compound events, several lines of effort are needed for future studies. A compound perspective on extremes (e.g., sand storms, saltwater intrusions) of different temporal and spatial scales is needed to further our understanding of recent extremes. In addition, the interaction of human activities (e.g., large inter-basin water transfer projects, intensive farming practices, massive reforestation efforts, and rapid urbanization) with the natural system in China should be considered in the modeling of compound events, which calls for the integration of physical drivers and societal drivers in understanding compound events. Moreover, the risk assessment of compound events should incorporate the dimension of exposure and vulnerability, especially in regions with large farm areas, fast urbanization, and dense and increasing population. This calls for enhanced cooperation between disciplines and sectors and updated methodology in estimating low-probability risk using large ensembles and evaluating model performance for simulating compound events. Last but not the least, the effectiveness of adaptation responses to multiple risks of compound events should be assessed by incorporating potentially compounding consequences of adaptation measures. As weather and climate extremes likely to increase with continued warming, further research on understanding and modeling compound events or extremes is essential to build resilience to climate change impacts in China.
